# Genome-Wide Analysis of NAC Transcription Factor Genes in the Invasive Weed *Mikania micrantha* Provides Insights into Potential Control Strategies

**DOI:** 10.3390/biology15110842

**Published:** 2026-05-28

**Authors:** Wenzheng Song, Yan’e Ding, Liying Yang, Weiwei Li, Ning Zhao

**Affiliations:** 1 College of Biological Science and Food Engineering, Southwest Forestry University, Kunming 650224, China; s978547680@gmail.com (W.S.); longnanding@163.com (Y.D.); 2 Yunnan International Joint Center of Urban Biodiversity, Kunming 650201, China; 15087150616@139.com; 3 Yunnan Key Laboratory of Biodiversity Information, Kunming Institute of Zoology, The Chinese Academy of Sciences, Kunming 650201, China

**Keywords:** *Mikania micrantha*, invasive plant control, NAC gene family, gene identification, bioinformatic analysis

## Abstract

*M. micrantha* is a highly destructive invasive weed causing severe ecological and economic damage in invaded regions. Conventional control methods remain insufficient, highlighting the need for targeted management strategies. NAC transcription factors, a plant-specific family, play key regulatory roles in growth, secondary metabolism, and stress responses. Here, we performed a genome-wide identification and characterization of the NAC gene family in *M. micrantha* using bioinformatic approaches based on *Arabidopsis thaliana* NAC sequences and the *M. micrantha* whole-genome assembly. A total of 76 *MmNAC* genes were identified from *M. micrantha*, and these members were classified into 13 subfamilies and one unclassified clade, with subdomains C and D being the most conserved. Five conserved motifs were identified; motifs 1 and 2 were present in over 94% of members. Chromosomal mapping showed 68 genes distributed unevenly across 18 of 19 chromosomes. Tissue expression analysis revealed that NAP and AtNAC3 subfamilies are highly expressed in seeds, while NAM and NAC2 subfamilies predominate in floral tissues, suggesting potential subfamily–tissue expression patterns. These findings provide a basis for understanding NAC functions in *M. micrantha* and identifying targets for its control.

## Simple Summary

*Mikania micrantha*, a highly invasive weed, has been shown to cause major damage to agriculture and ecosystems on a global scale. Conventional control methods have proven ineffective due to the plant’s prolific flowering and seed production. This study identified and characterized 76 NAC transcription factor genes in its genome, which were then grouped into a total of 13 evolutionary clades. The analysis revealed that genes with particularly high levels of expression in flowers and seeds may be tentatively linked to reproductive regulation. This finding indicates that these genes could potentially play certain roles in the weed’s rapid reproductive cycle and offer gene targets for silencing-based interventions, supporting the development of precise, eco-friendly biocontrol strategies.

## 1. Introduction

NAC transcription factors constitute one of the largest plant-specific transcription factor (TF) families, and their name is derived from three founding members, NAM [1], ATAF1/2, and CUC2 [2]. All members harbor a highly conserved NAC domain at the N-terminus, which can be subdivided into five subdomains designated A through E. Subdomain A participates in the formation of functional dimers [3], while subdomains B and E exhibit relatively weak conservation and are thought to contribute to functional diversification. In contrast, subdomains C and D are highly conserved: subdomain C is implicated in DNA binding, whereas subdomain D, in addition to its role in DNA recognition, contains both a nuclear localization signal and a repression domain [4,5,6]. The C-terminal region constitutes a highly variable transcriptional regulatory domain capable of either activating or repressing gene expression [7]. Based on the sequence characteristics of the NAC domain, Ooka et al. classified the NAC family into two major groups (Groups I and II) comprising 18 subfamilies [8]. As plant-specific TFs, NAC proteins regulate gene expression positively or negatively by binding to cis-acting elements in target gene promoters, thereby participating in diverse processes of plant growth, development, and stress responses [9,10,11].

NAC TFs have been shown to participate extensively in plant responses to diverse biotic and abiotic stresses as well as hormonal signaling pathways [11,12]. In rice, *OsNAC5*, *OsNAC9*, and *OsNAC10* are induced by drought, high salinity, and abscisic acid (ABA) and enhance stress tolerance by modulating the expression of downstream stress-responsive genes [13]. Beyond stress responses, NAC TFs fulfill critical roles in plant growth and development, particularly in the regulation of floral organ development and flowering time [14]. *Arabidopsis* overexpressing *ANAC092* shows enhanced expression of pollen development-related genes [15], while *CUC1* and *CUC2* are required for stamen and pistil development, and their loss-of-function mutations can result in sterility or abnormal ovule formation [16]. In terms of flowering regulation, NAC TFs modulate downstream floral integrator genes in response to environmental cues such as temperature and light, as well as to endogenous signals including plant age and phytohormones, thereby controlling flowering time. Additionally, NAC family members are broadly involved in leaf senescence [17,18,19], fruit ripening [20], lateral root development [21], and seed germination [22]. Although the NAC TF functions have been extensively characterized in model plants and various crops, their roles in the Asteraceae species *M. micrantha* remain largely unexplored.

*M. micrantha* is a perennial herbaceous vine belonging to the family Asteraceae, native to Central and South America. Owing to its exceptional growth rate and reproductive capacity, it has invaded more than 70 countries across Southeast Asia, the South Pacific, and Africa [23], making it one of the most destructive invasive weeds in tropical and subtropical regions worldwide [24]. *M. micrantha* can grow at an average rate of 8–9 cm per day, propagate vegetatively through rooting at stem nodes, and produce large quantities of pappus-bearing seeds. These characteristics have earned it the colloquial name “mile-a-minute weed” [25]. *M. micrantha* has been observed to cause substantial ecological and agricultural damage in invaded regions via two principal mechanisms: physical shading through dense canopy formation and allelopathic inhibition of neighboring vegetation [26,27]. These impacts translate into considerable economic consequences, including reductions in rubber seed germination and oil palm yield in Malaysia [28], with annual losses across Southeast Asian woody cash crops estimated to exceed USD 2 billion [29]. The International Union for Conservation of Nature (IUCN) has classified *M. micrantha* as one of the “100 of the World’s Worst Invasive Alien Species” in acknowledgement of its destructive capacity [30].

Conventional management approaches, including manual removal, chemical treatment, and classical biological control, have proven inadequate to suppress the spread of *M. micrantha* at its source [31,32,33]. This limitation is largely attributable to its vigorous sexual reproductive capacity; a single stalk of *M. micrantha* can produce 20,000–40,000 seeds in one season [34]. Therefore, a thorough understanding of the floral and reproductive regulatory mechanisms of *M. micrantha* and the identification of key regulatory targets are essential for developing effective and sustainable management strategies. The NAC gene family plays a crucial role in plant growth and development, particularly in regulating flowering. A systematic analysis of the *MmNAC* gene family, focusing on members associated with key reproductive processes such as flowering and seed development, will help to elucidate how *M. micrantha* produces large quantities of seeds for reproduction within a short period of time. This will provide a molecular foundation for control strategies related to gene silencing and RNA interference.

In this study, we performed genome-wide identification and comprehensive bioinformatic analyses of the NAC gene family in *M. micrantha*, encompassing protein physicochemical properties, conserved motifs, gene structure, chromosomal localization, and phylogenetic relationships. We further analyzed the expression profiles of *MmNAC* genes across different tissues and developmental stages of floral organs using transcriptome data. This study contributes to a better understanding of NAC TF functions in *M. micrantha* and supports the identification of molecular targets relevant to controlling its invasion.

## 2. Materials and Methods

### 2.1. Identification of the NAC Gene Family in M. micrantha

Two complementary strategies were integrated—homology-based BLAST searching and Hidden Markov Model (HMM) profiling—to systematically identify members of the NAC gene family in *M. micrantha*. First, the whole-genome assembly of *M. micrantha* (accession number: GCA_009363875.1) was retrieved from the NCBI database (https://www.ncbi.nlm.nih.gov/, accessed on 3 March 2026), and its coding sequences (CDS) and protein sequences were extracted using TBtools-II, v2.453.

For the homology search, the *Helianthus annuus* NAC sequences were used as queries to perform a local BLASTp search against the entire *M. micrantha* protein database using the BLAST GUI Wrapper integrated in TBtools-II, v2.453 (the BLAST settings were left at their default values: E-value < 1 × 10^−5^; NumOfHits = 500; and NumOfAligns = 250), yielding a preliminary set of candidate sequences. For a more comprehensive identification, the HMM profile for the NAC domain (PF02365) was downloaded from the Pfam database (http://pfam.xfam.org/, accessed on 4 March 2026), and HMMER 3.0 was used to search the *M. micrantha* protein database with an E-value threshold of <1 × 10^−5^. Retaining only candidates identified by both methods minimizes the false positives inherent in either approach alone, thereby providing a more rigorous criterion for family membership.

After merging and de-replicating candidate sequences from both methods, the resulting sequences were submitted to the NCBI Batch CD-Search tool (https://www.ncbi.nlm.nih.gov/Structure/bwrpsb/bwrpsb.cgi, accessed on 5 March 2026) and the SMART online platform (https://smart.embl.de, accessed on 5 March 2026) for conserved domain verification using default parameters. Sequences lacking a complete NAC domain were manually removed.

### 2.2. Prediction of Physicochemical Properties

The amino acid sequence length (aa), theoretical molecular weight (MW), isoelectric point (pI), instability index, aliphatic index, and grand average of hydropathicity (GRAVY) were computed for each protein using the ExPASy ProtParam tool (https://web.Expasy.org/protparam/, accessed on 6 March 2026). Subcellular localization was predicted by WoLF PSORT (https://www.genscript.com/wolf-psort.html, accessed on 6 March 2026).

### 2.3. Phylogenetic Analysis

Multiple sequence alignment of NAC protein sequences from *M. micrantha* and several other Asteraceae species was performed using MUSCLE in MEGA 11 with default parameters. The phylogenetic tree was then reconstructed using the Neighbor-Joining (NJ) method with 1000 bootstrap replicates, the Jones–Taylor–Thornton (JTT) amino acid substitution model, and a site coverage cutoff of 95%. The resulting NWK file was visualized and refined using the online tool iTOL (https://itol.embl.de/, accessed on 15 March 2026).

### 2.4. Conserved Domain and Motif Analysis

Multiple sequence alignment results from Section 2.3 were visualized using ESPript v3.2 (https://espript.ibcp.fr/ESPript/cgi-bin/ESPript.cgi, accessed on 16 March 2026) to facilitate identification of conserved domains. The procedure of conserved motif analysis was executed by employing the MEME Suite (https://meme-suite.org/meme/tools/meme, accessed on 16 March 2026). The maximum number of motifs was set to five, the motif width was designated as six to fifty amino acids, and all other parameters were set to their default values [35]. The resulting XML file was imported into TBtools-II, v2.453, for visualization [36].

### 2.5. Chromosomal Localization and Gene Structure Analysis

Chromosomal localization and gene structure of *MmNAC* members were analyzed based on the genome annotation file in GFF3 format (GCA_009363875.1) using TBtools-II, v2.453. The Gene Location Visualize function was used to extract the chromosomal coordinates (chromosome number, start position, and end position) of each *MmNAC* gene and to generate the chromosomal distribution map. Exon–intron structures were extracted and visualized using the Gene Structure View function in TBtools-II, v2.453.

### 2.6. Tissue-Specific Expression Analysis

Transcriptome datasets for five tissues: root (SRR10596657), stem (SRR10596656), leaf (SRR10596655), flower (SRR10596654), and seed (SRR10596653), were retrieved from the NCBI SRA database (https://www.ncbi.nlm.nih.gov/sra, accessed on 25 March 2026) [37]. The generation of this whole-transcriptome dataset occurred on Neilingding Island, Shenzhen, Guangdong Province, China, through the utilization of PacBio SMRT and Illumina RNA-Seq technologies. The TPM values of all *MmNAC* transcripts were evaluated by the Kallisto Super GUI Wrapper plugin within the TBtools-II, v2.453, and heatmaps were generated based on the TPM values by TBtools-II, v2.453.

## 3. Results

### 3.1. Identification and Physicochemical Characterization of the MmNAC Gene Family

Utilizing the HMM and BLAST methodologies, and following the intersection process and the removal of sequences that were insufficiently lengthy or lacked complete domains, a total of 76 members of the *MmNAC* TF family were identified in *M. micrantha* (Table S1). The number of members in the *M. micrantha* NAC TF family is lower than the average for dicotyledons (125) but closer to the average for gymnosperms (75). By way of contrast, the *H*. *annuus*, which also belongs to the Asteraceae family, has only 21 members in its NAC TF family [38]. Comprehensive physicochemical characterization of the 76 *MmNAC* proteins revealed considerable diversity across multiple parameters (Table S2). Protein lengths ranged from 189 to 636 amino acids (aa), with corresponding theoretical molecular weights (MW) spanning from 21.83 to 72.11 kDa. The theoretical isoelectric points (pI) exhibited a broad distribution, ranging from 4.40 to 9.57, indicating substantial variation in the surface charge properties of *MmNAC* proteins. Notably, 70% of *MmNAC* members were predicted to be acidic proteins, suggesting that *MmNAC* proteins are predominantly acidic in nature, indicative of functional and biochemical diversity within this family.

The subcellular localization of the 76 *MmNAC* proteins was predicted using WoLF PSORT, revealing a predominant nuclear distribution consistent with their roles as TFs (Table S3). Of the 76 members, 75 (98.7%) were predicted to localize primarily in the nucleus, thereby supporting their function as transcriptional regulators that bind to DNA cis-regulatory elements to modulate downstream gene expression. A subset of 53 members (69.7%) was predicted to localize in the cytoplasm, 46 members (60.5%) were predicted to reside in the chloroplast, and 21 (27.6%) in the peroxisome. Membrane-associated members constituted 26 individuals, representing 34.2% of the total. The prediction indicated that 22 (28.9%) members would localize in the mitochondria. The diverse subcellular distribution of *MmNAC* proteins suggests that functional differentiation may exist within this family.

### 3.2. Conserved Domain Analysis of MmNAC Proteins

Multiple sequence alignment of the 76 *MmNAC* proteins using DNAMAN revealed that virtually all members contain the A and C canonical N-terminal conserved subdomains characteristic of NAC TFs. Only the ATAF subfamily lacks the A subdomain, whilst the ONAC003 subfamily lacks the C subdomain; the B and D subdomains are frequently absent or incomplete. Subfamilies lacking the B subdomain include NAM, NAC1, OsNAC7, TERN, NAC2, ONAC003 and SENU5, whilst subfamilies lacking the D subdomain include NAM, NAC1, OsNAC7, NAC2, ATAF, OsNAC3 and ONAC003. In addition, the E subdomain is also missing in genes from multiple subfamilies (Table S4 and Figures S1–S13). We therefore infer that the A and C subdomains are more conserved than the other three subdomains.

### 3.3. Phylogenetic Analysis and Subfamily Classification

**Figure 1:**
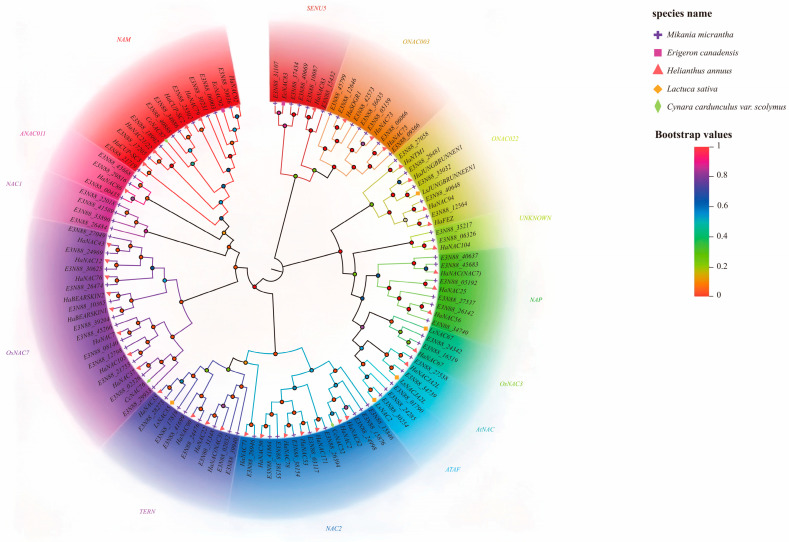
Phylogenetic tree of the NAC gene family in *M. micrantha*. The tree was constructed using the Neighbor-Joining method with 1000 bootstrap replicates based on NAC protein sequences from *M. micrantha* and selected Asteraceae species.

A phylogenetic tree was constructed using the 76 MmNAC proteins together with NAC sequences from four closely related Asteraceae species retrieved from NCBI, including *H. annuus* (39), *Cynara cardunculus var. Scolymus* (3), *Lactuca sativa* (5), and *Erigeron canadensis* (2) (Figure 1). Following the classification system of Ooka et al. and using N-terminal domain sequence features as the criterion, all 125 NAC genes were assigned to 13 subfamilies and one unclassified clade [8]. The 13 subfamilies are: TERN, SENU5, NAP, AtNAC3, ATAF, OsNAC3, NAC2, ANAC011, ONAC022, OsNAC7, NAC1, NAM, and ONAC003. Three genes, E3N88_35217, E3N88_06326 and *HaNAC104*, could not be assigned to any established subfamily. It should be noted that, as is commonly observed in phylogenetic analyses of large plant TF gene families, some internal nodes in the tree exhibit relatively low bootstrap support values; this is a recognized limitation of the standard bootstrap method when applied to datasets comprising large numbers of sequences [39], and subfamily assignments were therefore corroborated by conserved motif composition and gene structure analyses as described below.

Each of the 14 clades contained between 3 and 16 genes and comprised members from at least two different species. The 76 *MmNAC* genes were distributed across all 14 clades, with per-clade membership ranging from 2 to 12. The OsNAC7, NAM, NAC2, and ONAC003 subfamilies contained the largest numbers of *MmNAC* members, whereas the NAP, SENU5, TERN, NAC1, ONAC022, ANAC011, OsNAC3, AtNAC3, ATAF, and unclassified clades were represented by fewer members. Furthermore, within the NAM, OsNAC7 and TERN subfamilies, analysis of the homology between *M. micrantha* NAC genes and *H. annuus* NAC genes reveals a high degree of similarity, with the number of members being relatively similar. However, in contrast to *M. micrantha*, *H. annuus* lacks members of the NAC1 and ATAF subfamilies. This disparity may reflect lineage-specific gene-loss patterns during evolution. The variable distribution of NAC gene copies from different species across subfamilies indicates that *M. micrantha* NAC genes share high sequence similarity with those of other Asteraceae species, suggesting putative orthologous relationships.

### 3.4. Conserved Motif and Gene Structure Analysis

**Figure 2:**
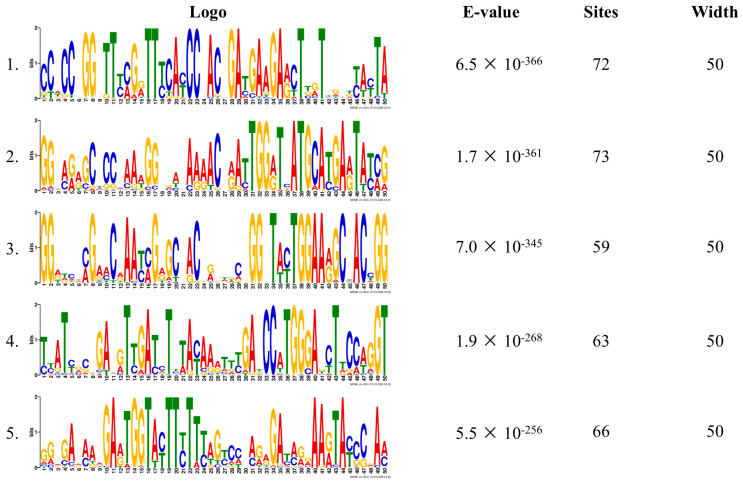
Conserved motif analysis of the NAC TF family in *M. micrantha*.

A total of five highly conserved motifs were identified (Figure 2). The E-values of all five motifs ranged from 6.5 × 10^−366^ to 5.5 × 10^−256^, indicating extreme statistical significance; each motif was 50 amino acids in length. Coverage differed among motifs: The presence of Motif 1 (74 genes; 97.3%) and Motif 2 (76 genes; 100.0%) was observed in the vast majority of family members and are therefore considered core conserved motifs, while Motif 5 (71 genes; 93.4%), Motif 3 (70 genes; 92.1%), and Motif 4 (70 genes; 92.1%) showed lower coverage with subfamily-specific distribution patterns. Sequence alignment demonstrated that these five motifs correspond to the conserved subdomains A through E of the NAM domain, respectively.

When examined in the context of the phylogenetic subfamily classification, genes within the same subfamily displayed highly consistent motif compositions, combinations, and arrangements, whereas marked differences existed between subfamilies. Members of the larger subfamilies, such as OsNAC7 and NAM, predominantly harbor all five motifs, representing a complete motif complement. The SENU5 and related small subfamilies exhibit their own distinctive motif combination patterns. These findings are consistent with the conclusions of Ooka et al. and confirm that motif composition can serve as an informative auxiliary criterion for NAC subfamily classification. Furthermore, these combinatorial patterns are the products of long-term evolutionary divergence, and the cooperative action of different motifs likely drives the structural and functional diversification of NAC proteins across subfamilies.

**Figure 3:**
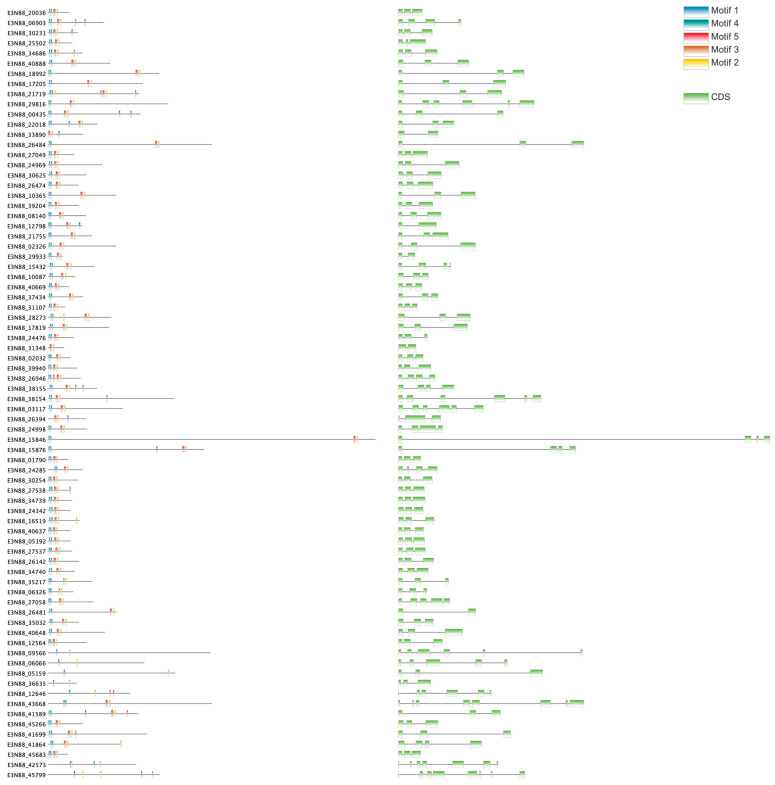
Gene structure analysis of NAC TFs in *M. micrantha*. (**Left**) Conserved motif distribution; (**Right**) intron–exon structure.

Gene structure analysis of the 76 *MmNAC* genes (Figure 3) revealed that all genes contain a coding sequence. Most genes were ≤3000 bp in length, while a minority exceeded 6000 bp. All genes contain 2–8 introns. Strikingly, genes belonging to the same or closely related subfamilies shared highly similar intron–exon structures and motif combination patterns, further validating the accuracy of the subfamily classification reported in this study.

### 3.5. Chromosomal Localization of MmNAC Genes

**Figure 4:**
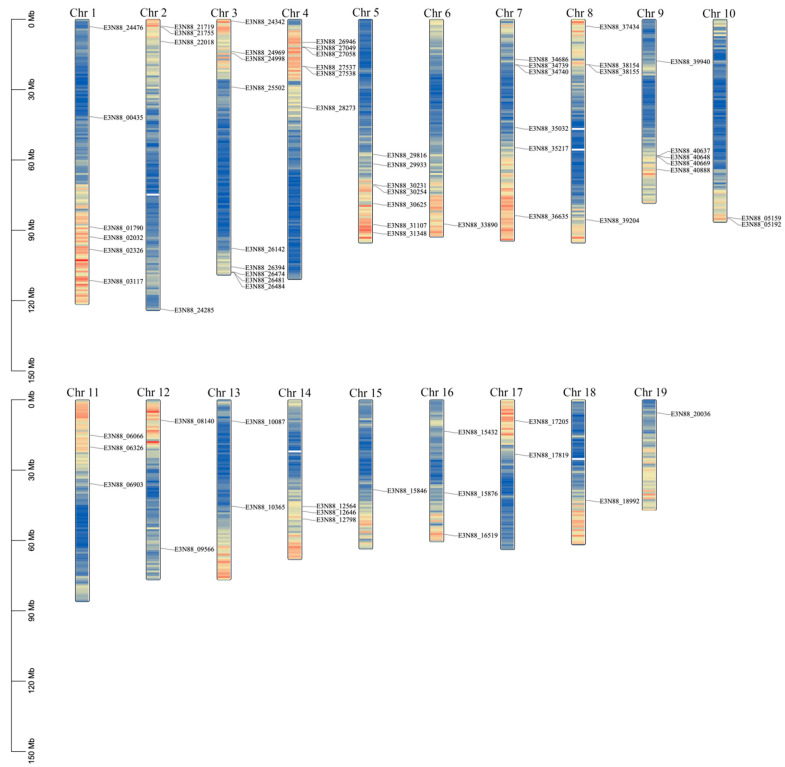
Chromosomal localization of *MmNAC* genes in *M. micrantha*. The color gradient along each chromosome represents gene density, ranging from blue (low gene density) to red (high gene density).

Chromosomal localization of the 76 identified *MmNAC* genes revealed that 68 genes are unevenly distributed across 18 of the 19 chromosomes, while the remaining 8 genes could not be assigned to a specific chromosome (Figure 4, Table S5). Chromosome 3 harbors the greatest number of *MmNAC* genes, followed by chromosomes 1, 4, 5, and 7, each carrying 6–7 genes. Chromosomes 15, 18, and 19 each contain only one *MmNAC* gene, while the remaining 11 chromosomes carry 2–5 genes each.

Notably, *MmNAC* genes exhibit a non-random chromosomal distribution. The majority of genes are located in the telomeric and sub-telomeric regions of chromosomes, with only a few located near centromeres. In addition, several chromosomes display tightly clustered gene pairs or small gene arrays, while most genes occur as singletons or are dispersed across distinct genomic regions.

### 3.6. Tissue-Specific Expression Analysis of MmNAC Genes

**Figure 5:**
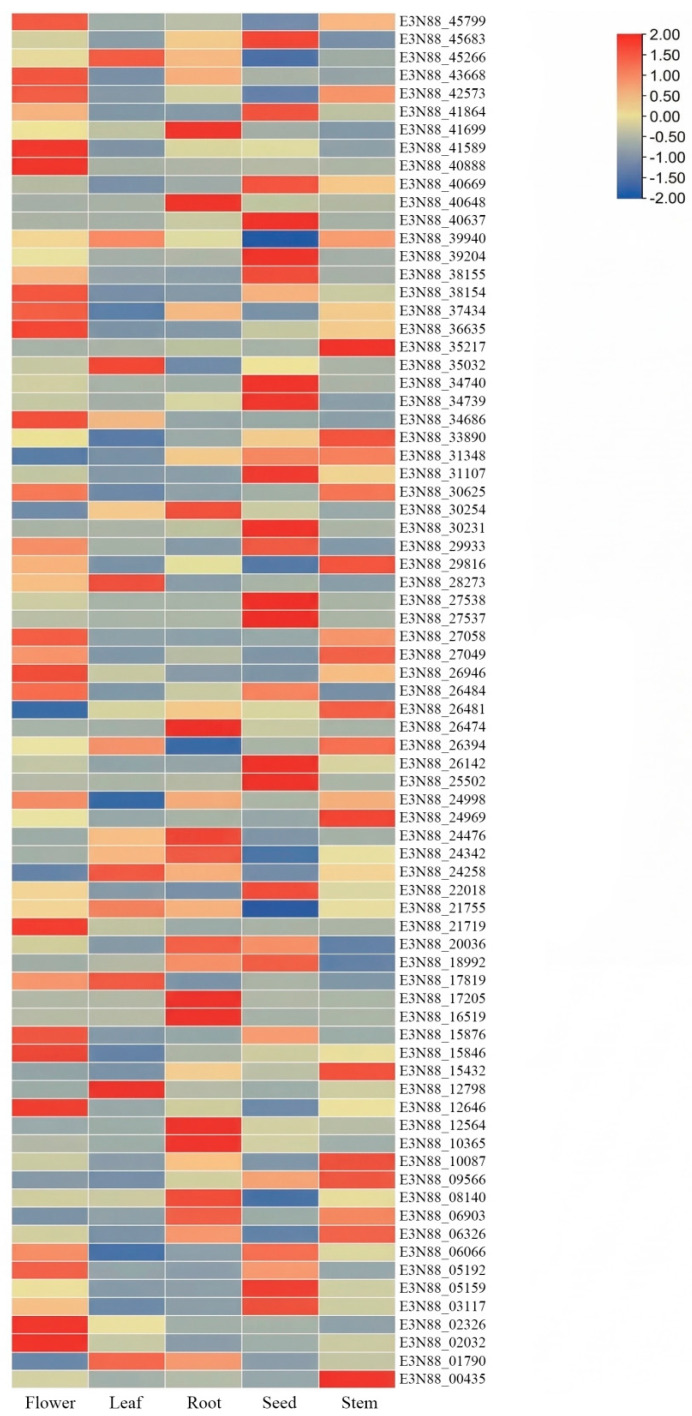
Heatmap of *MmNAC* gene family expression in different tissues of *M. micrantha*. The color scale represents normalized expression values (TPM); red indicates high expression, and blue indicates low expression.

Transcriptome data were used to analyze expression profiles of *MmNAC* genes in five tissues: root, stem, leaf, flower, and seed (Tables S6 and S7). Expression heatmaps were constructed using TBtools-II (Figure 5).

The results indicated that *MmNAC* genes are expressed in all examined tissues. In seeds, 19 genes were highly expressed, predominantly from the NAP and AtNAC3 subfamilies (E3N88_40637 and E3N88_34740). In flowers, 17 genes were highly expressed, concentrated in the NAM and NAC2 subfamilies (E3N88_40888 and E3N88_34686). Roots and stems contained fewer highly expressed genes; root-expressed genes were mainly from the OsNAC3 subfamily, whereas stem-expressed genes were primarily from the ANAC011 subfamily. Leaf tissue exhibited predominantly low expression levels across most family members.

Notably, genes within the same subfamily shared highly conserved expression patterns: NAP/AtNAC3 showed preference for seeds, NAM/NAC2 were concentrated in flowers, OsNAC3 was predominantly expressed in roots, and ANAC011 was dominant in stems.

## 4. Discussion

The 76 NAC TFs identified in *M. micrantha* represent a moderately sized family compared with those reported in other plant species to date. Previous genome-wide analyses have reported substantial variation in NAC family size across the plant kingdom, ranging from as few as 9 members in the liverwort Marchantia polymorpha to as many as 410 in the allotetraploid Brassica napus [38]. Notably, the number of NAC genes identified in *M. micrantha* falls below the reported mean for dicotyledonous plants (125), despite monocots exhibiting a higher average complement (141). Compared with the members of the NAC TF family in the model plant of the Asteraceae family, the *H*. *annuus* (21), the NAC TF family in *M. micrantha* has undergone significant expansion. Gene duplication is one of the key evolutionary processes that has driven diversification within the NAC family of angiosperms. Rates of gene duplication and loss vary across subfamilies, and relaxed selection has driven functional diversification [40]. This hypothesis may also be relevant given that *M. micrantha* possesses members of the NAC1 and ATAF subfamilies, which are absent in *H*. *annuus*.

The results of subcellular localization predictions for *MmNAC* proteins reveal a diverse pattern of subcellular distribution. The results of the study indicated that 75 members (98.7%) were predicted to localize primarily in the nucleus, which is highly consistent with the classical function of NAC TFs in regulating the expression of downstream genes through binding to DNA cis-regulatory elements. This finding serves to further confirm the role of the *MmNAC* family as nuclear-localized transcription regulators. It is noteworthy that an additional 53 members (69.7%) were also predicted to localize to the cytoplasm. As has been established by earlier research, there is evidence to suggest that certain NAC proteins exist as membrane-bound, transcriptionally dormant precursors. In response to developmental or stress signals, these precursors undergo proteolytic cleavage, releasing transcriptionally active fragments. These fragments are subsequently transported to the nucleus, where they exert their function. Based on in silico predictions, *MmNAC* proteins exhibiting cytoplasmic localization signals might exert their transcriptional regulatory functions by this process, a possibility that warrants further experimental validation [41,42]. Furthermore, some members of the *MmNAC* family have been localized to regions such as chloroplasts, peroxisomes and the plasma membrane. The diverse subcellular distribution patterns exhibited by members of the *MmNAC* family are indicative of highly specialized functions.

Based on the subdomain characteristics proposed by Ooka et al. [8], we found that different subfamilies within the NAC TF family exhibit distinct subdomain characteristics; specifically, subdomains A and C are relatively conserved, whereas subdomains B, D and E are more variable. Previous studies have indicated that subdomain C may be involved in DNA binding [43] and that subdomains C and/or D within the same subfamily may jointly participate in the recognition of the same cis-acting element [38]. It can therefore be inferred that if the absence of subdomains C and D affects the binding of subfamily members to DNA, this may impair their ability to function as TFs. Furthermore, members of the NAC1, NAC2 and OsNAC7 subfamilies exhibit analogous subdomain characteristics, whereas members of the ANAC011, AtNAC3 and NAP subfamilies are characterized by the presence of all five subdomains.

In this study, the MEME suite was utilized to identify motifs in 76 members of the *M. micrantha* NAC TF family, resulting in the identification of five high-confidence motifs. A total of 66 members were found to contain all five motifs, while motifs 1 and 2 were present in almost every member. Notably, motifs 1 and 2 were absent only in E3N88_31348 and E3N88_33890, respectively. E3N88_09566, E3N88_06066, E3N88_05159 and E3N88_36635 were found to belong to the ONAC003 subfamily. Despite the identification of only two motifs in these sequences, it is noteworthy that they contain complete copies of Motif 1 and Motif 2. This finding underscores the conservation and central importance of these two motifs. Notably, motifs 1 and 4 are usually found in close proximity to each other and exhibit a relatively fixed spacing, manifesting in conjunction at the 5′ terminus of the sequence. In a manner analogous to the aforementioned observations, motifs 2 and 5 manifest in pairs in a configuration analogous to that observed near the 3′ terminus of the sequence. It has been hypothesized that the subfamily-specific motif combinations observed here likely reflect functional divergence among NAC subgroups. The C-terminal region of NAC proteins contains group-specific conserved motifs that are shared within a subfamily but vary considerably across different subfamilies. Consistently, NAC subfamily-specific accessory motifs have been identified in *Citrullus lanatus*, where unique motif combinations have been proposed to underlie the distinct biological functions of individual subfamilies [44]. The fixed positional arrangement of motif pairs 1/4 and 2/5 at the 5′ and 3′ termini, respectively, may therefore reflect structural constraints required for cooperative functions such as dimerization or co-regulator recruitment that are specific to particular NAC subfamilies [45].

Genes involved in distinct biological processes typically exhibit differential expression across various tissues and developmental stages, and these expression profiles serve as a significant indicator of their functional roles in plant growth and development, organ formation, and environmental responses [46]. This study utilized transcriptomic data analysis to characterize the expression patterns of 76 *MmNAC* genes across five core tissues of *M. micrantha*, namely roots, stems, leaves, flowers and seeds. Although *MmNAC* genes were broadly expressed across all five examined tissues, seeds and flowers harbored the greatest number of highly expressed members. Among these, 19 *MmNAC* genes showing elevated expression in seeds are predominantly affiliated with the NAP, NAC2, and NAM subfamilies. This is consistent with findings in rice, where downregulation of *OsNAP* expression effectively promotes grain filling, thereby significantly increasing crop yield [47]. In *Arabidopsis thaliana*, *NAC2* and *NAM* expression are confined to the outer integument of ovules, exhibiting a high degree of tissue specificity [48]. It is therefore speculated that the highly expressed genes of the NAP, NAC2, and NAM subfamilies in *M*. *micrantha* may participate in the regulation of seed maturation, dormancy, and germination by modulating the expression of key genes involved in seed development.

In *M. micrantha*, we focused particularly on the flowering-related NAC genes. The 17 highly expressed *MmNAC* genes in floral tissues belong mainly to the NAM and NAC2 subfamilies, which is consistent with previous reports on the roles of NAC genes in plant floral organ development, flowering regulation and pollen development. For example, in transgenic *Arabidopsis*, *PwNAC2* delays flowering by altering the expression of *FT*, *SOC1* and *FLC* [49], whilst the overexpression of *OsNAC2* in rice also delays flowering [50]. Some genes in the *NAC2* subfamily are also involved in hormone signaling during floral development, influencing the normal development of floral organs. For instance, in cucumber, *CsNAC2* is specifically expressed in female floral organs, whilst its heterotopic expression leads to carpelization of stamens and reduced ethylene accumulation, indicating that the NAC2 subfamily participates in the regulation of normal floral organ development via the ethylene signaling pathway [51]. As one of the most ancient subfamilies within the NAC gene family, members of the NAM subfamily regulate the morphogenesis of floral organs such as sepals and petals in various plants. In *Arabidopsis*, functional loss of key members of this subfamily, *CUC1* and *CUC2*, leads to the fusion of sepals and stamens [52]. Given that *M. micrantha* is characterized by a long flowering period and high seed-setting rate [53], further analysis and functional validation of highly expressed genes in the NAM/NAC2 subfamilies, such as E3N88_40888 and E3N88_34686, to determine their roles in flowering regulation and floral morphogenesis may provide a theoretical basis for future control of *M. micrantha* using RNAi technology.

In contrast to the high levels of expression observed in seeds and flowers, fewer *MmNAC* genes exhibited significant levels of expression in root and stem tissues. The expression of these genes was predominantly concentrated in the OsNAC3 (3 genes) and OsNAC7 (2 genes) subfamilies, respectively. Previous studies have demonstrated that the OsNAC3 subfamily is involved in root growth and development as well as stress-responsive regulation in rice [54], whereas the OsNAC7 subfamily participates in the regulation of root and stem growth [55]. As a climbing invasive plant, *M. micrantha* requires rapid root adaptation to heterogeneous soil environments, while its stems must possess considerable mechanical strength to support climbing growth. Accordingly, OsNAC3 subfamily genes, highly expressed in roots, may enhance habitat adaptability through stress-responsive regulation, whereas OsNAC7 subfamily genes, highly expressed in stems, may optimize climbing characteristics by promoting lignification processes. Together, these two mechanisms may constitute the molecular basis underlying the robust invasive capacity of *M. micrantha*.

The majority of *MmNAC* genes exhibited low expression levels in leaves, which contrasts with the high leaf expression patterns reported for certain NAC genes in *Arabidopsis* [56], wheat [57], and other plants. These discrepancies suggest that, through long evolutionary divergence, even orthologous genes derived from a common ancestor can acquire divergent expression patterns and functions, likely in response to selection pressures favoring improved environmental adaptability and reproductive fitness [58,59]. Functionally beneficial genes tend to be reinforced, while less critical genes are attenuated or replaced; such shifts are also observed among individuals within the same species [60]. Therefore, although NAC TFs are conserved at the family level, their functional roles can vary considerably across plant species. As this expression analysis is descriptive in nature, the associated functional interpretations are putative and await empirical validation.

Taken together, this work provides evidence of both conserved and species-specific roles of NAC TFs in *M. micrantha*, improving our understanding of their contribution to its growth, development, and invasiveness.

## 5. Conclusions

In this study, 76 NAC TF genes were identified from the whole genome of *M. micrantha*, and their physicochemical properties, structural features, evolutionary relationships, and tissue expression profiles were systematically analyzed. These genes were classified into 13 subfamilies; OsNAC7, NAM, NAC2, and ONAC003 were the dominant subfamilies. Motif 1 and Motif 2 represent the core conserved motifs, and both motif composition and gene structure are highly conserved within individual subfamilies. Chromosomal mapping of the NAC gene family in *M. micrantha* generated a physical genomic map, providing a foundation for analyzing tandem repeats within the family. Tissue expression analysis revealed that certain members of the NAP/AtNAC3 subfamily show notably higher expression levels in seeds, while members of the NAM/NAC2 subfamily tend to be more highly expressed in flowers. Similarly, several OsNAC3 subfamily members show elevated expression in roots, and a subset of ANAC011 subfamily members display higher expression levels in stems.

Overall, this work contributes to a deeper understanding of NAC TFs in *M. micrantha*. In the future, further functional validation could be conducted using experimental methods such as heterologous overexpression of members of the NAM and NAC2 subfamilies, with a view to elucidating their roles in flower regulation and providing a theoretical basis for the use of RNAi technology in the control of *M. micrantha*.

## Data Availability

The data that support the findings of this study are available in the Supplementary Material of this article.

## Supplementary Materials

The following supporting information can be downloaded at: https://www.mdpi.com/article/10.3390/biology15110842/s1, Figure S1: Multiple sequence alignment of ANAC011 subfamily members; Figure S2: Multiple sequence alignment of ATAF subfamily members; Figure S3: Multiple sequence alignment of AtNAC3 subfamily members; Figure S4: Multiple sequence alignment of NAC1 subfamily members; Figure S5: Multiple sequence alignment of NAC2 subfamily members; Figure S6: Multiple sequence alignment of NAM subfamily members; Figure S7: Multiple sequence alignment of NAP subfamily members; Figure S8: Multiple sequence alignment of ONAC003 subfamily members; Figure S9: Multiple sequence alignment of ONAC022 subfamily members; Figure S10: Multiple sequence alignment of OsNAC3 subfamily members; Figure S11: Multiple sequence alignment of OsNAC7 subfamily members; Figure S12: Multiple sequence alignment of SENU5 subfamily members; Figure S13: Multiple sequence alignment of TERN subfamily members; Table S1: The protein sequences of 76 members of the *M. micrantha* NAC TF family; Table S2: Homology alignment of 76 members of the *M. micrantha* NAC TF family; Table S3: Prediction of subcellular localisation for 76 members of the *M. micrantha* NAC TF family; Table S4: Presence or absence of five conserved subdomains across 13 NAC transcription factor subfamilies in *M*. *micrantha*; Table S5: Chromosomal localisation of 76 *M. micrantha* NAC genes; Table S6: TPM values for the expression levels of 76 *M. micrantha* NAC genes in various tissues; Table S7: Count values for the expression levels of 76 *M. micrantha* NAC genes in various tissues.
