# Supplementary figures and images for: Genome-Wide Analysis of NAC Transcription Factor Genes in the Invasive Weed Mikania micrantha Provides Insights into Potential Control Strategies
Source: Biology (Basel). 2026 May 28;15(11):842. doi: 10.3390/biology15110842 (PMC13255735; doi:10.3390/biology15110842)

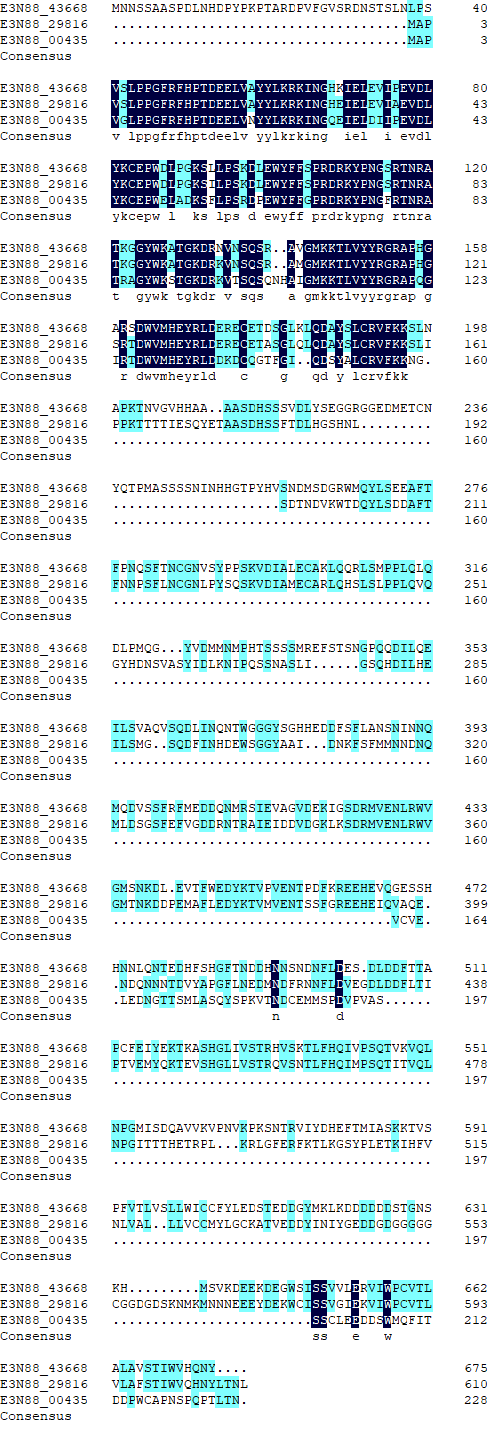

Supplement: Supplementary file 1 [file biology-15-00842-s001.zip › Figure S1.tif]

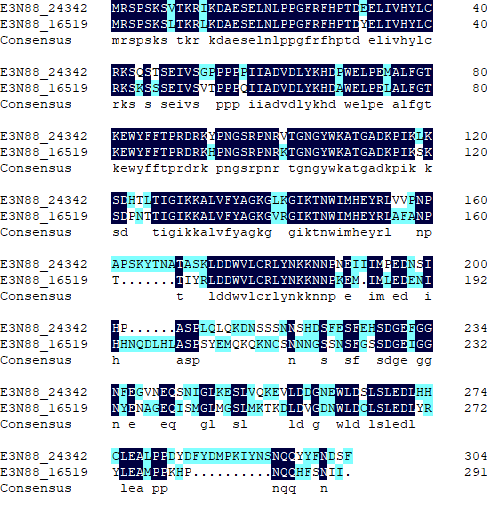

Supplement: Supplementary file 1 [file biology-15-00842-s001.zip › Figure S10.tif]

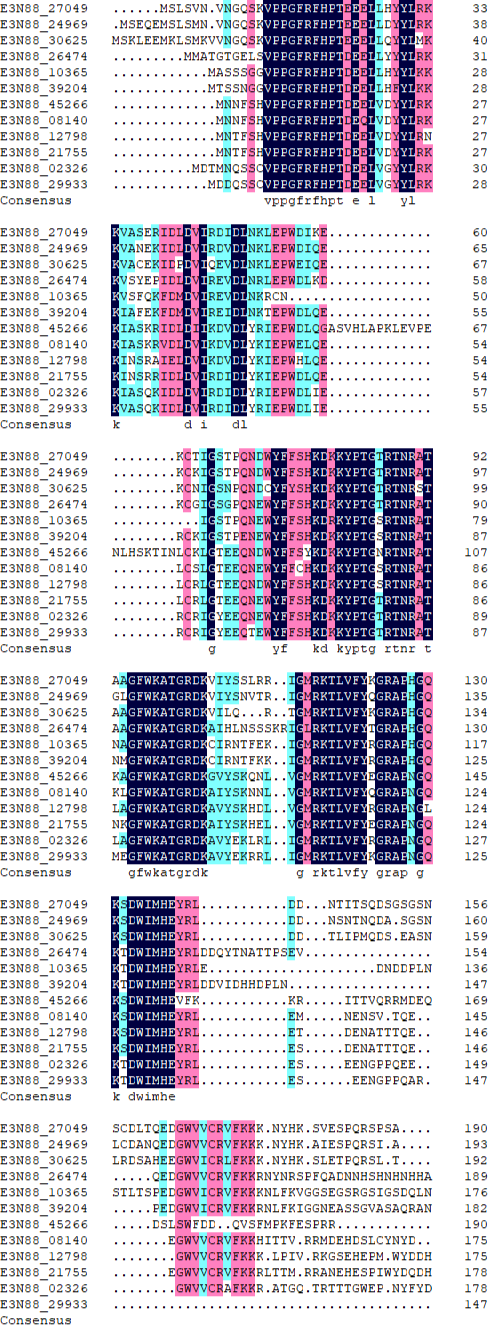

Supplement: Supplementary file 1 [file biology-15-00842-s001.zip › Figure S11.tif]

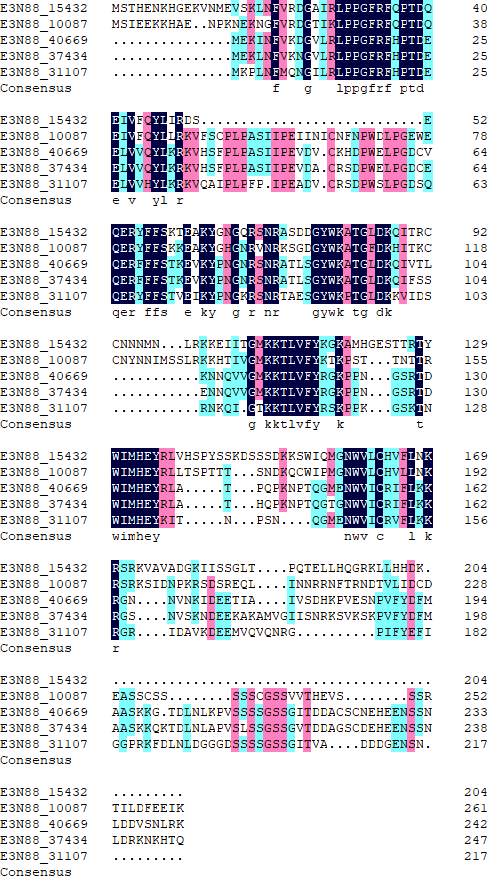

Supplement: Supplementary file 1 [file biology-15-00842-s001.zip › Figure S12.tif]

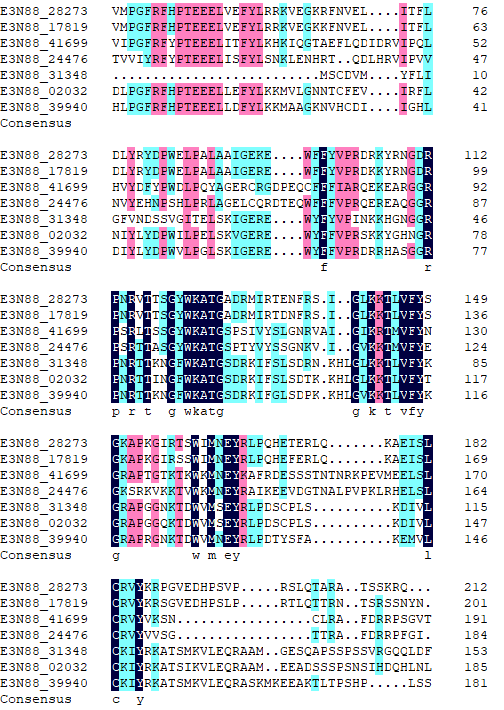

Supplement: Supplementary file 1 [file biology-15-00842-s001.zip › Figure S13.tif]

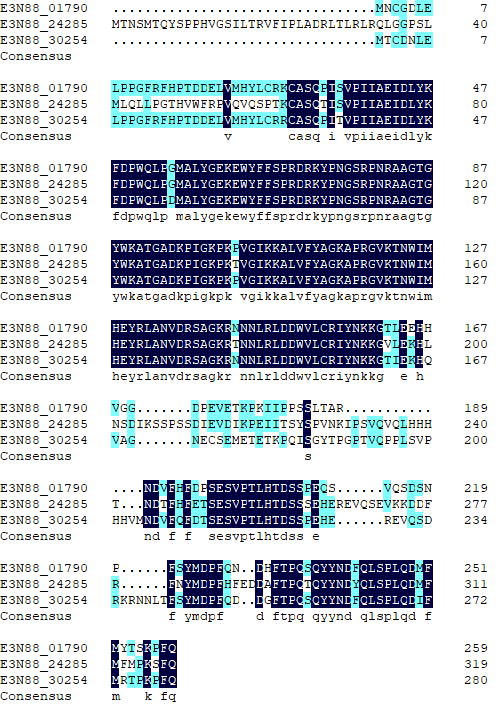

Supplement: Supplementary file 1 [file biology-15-00842-s001.zip › Figure S2.tif]

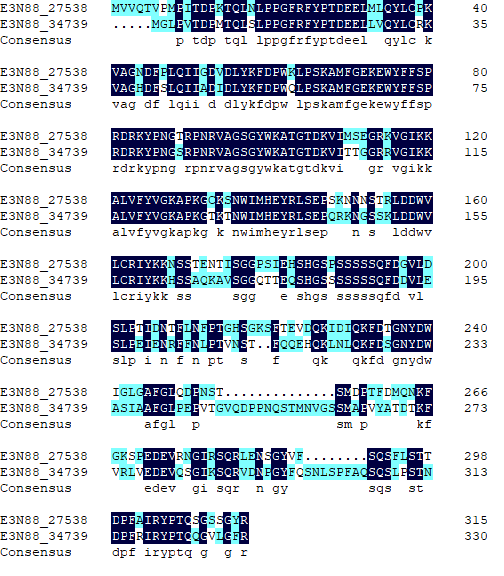

Supplement: Supplementary file 1 [file biology-15-00842-s001.zip › Figure S3.tif]

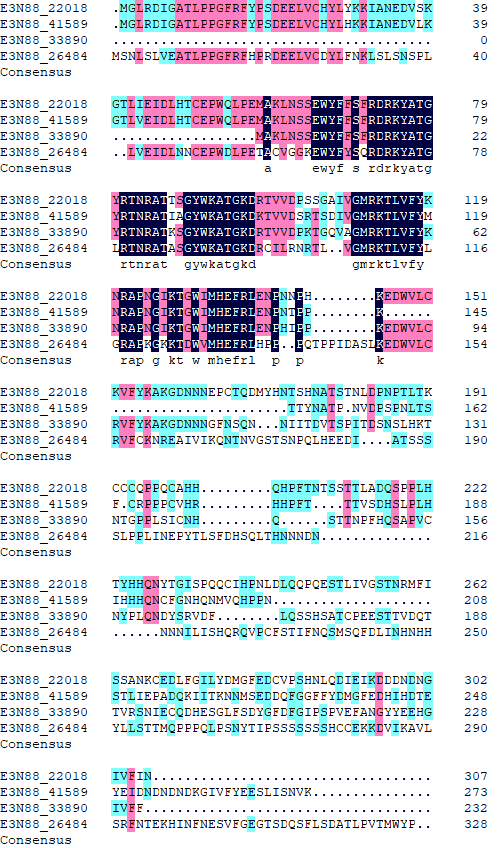

Supplement: Supplementary file 1 [file biology-15-00842-s001.zip › Figure S4.tif]

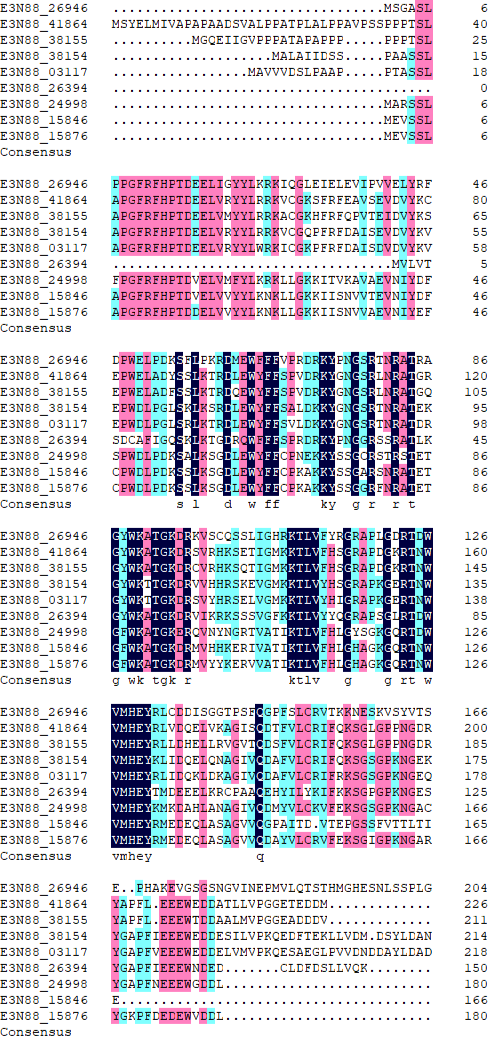

Supplement: Supplementary file 1 [file biology-15-00842-s001.zip › Figure S5.tif]

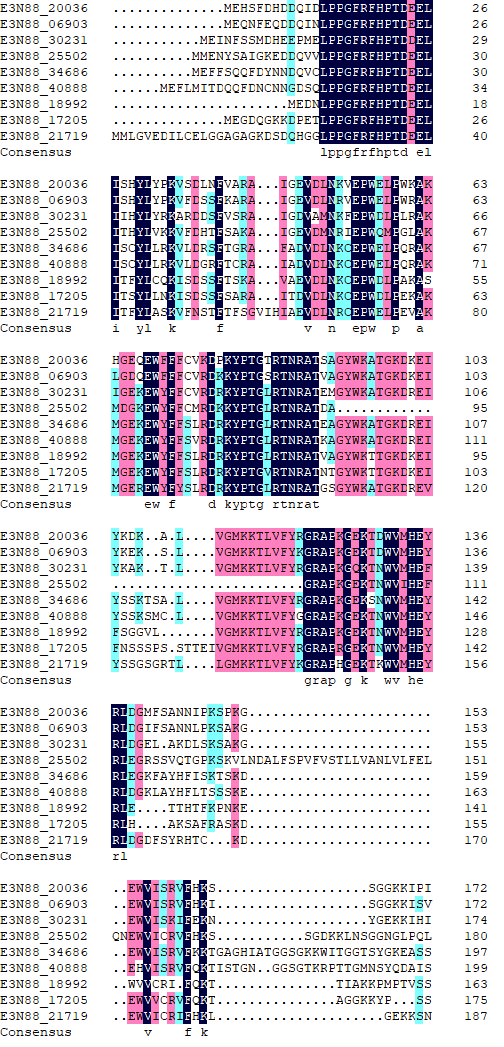

Supplement: Supplementary file 1 [file biology-15-00842-s001.zip › Figure S6.tif]

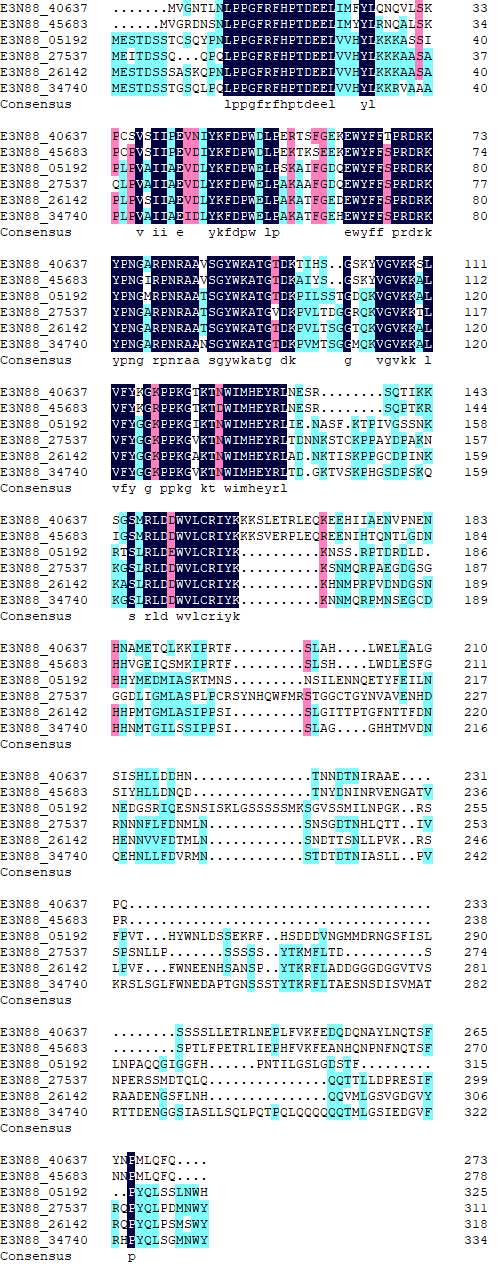

Supplement: Supplementary file 1 [file biology-15-00842-s001.zip › Figure S7.tif]

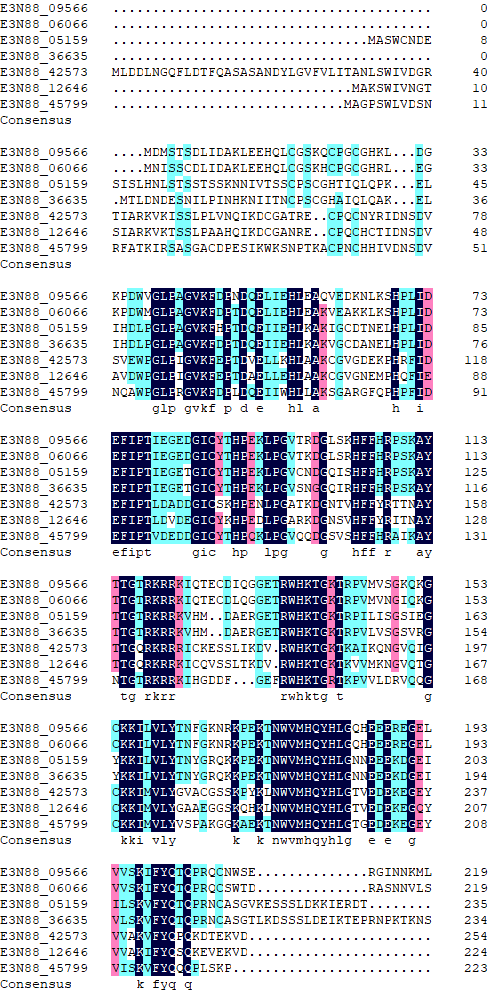

Supplement: Supplementary file 1 [file biology-15-00842-s001.zip › Figure S8.tif]

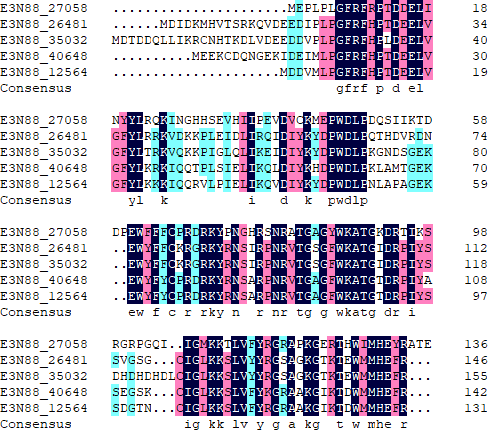

Supplement: Supplementary file 1 [file biology-15-00842-s001.zip › Figure S9.tif]
